# One of us or one of them? The effects of the model’s and observer’s characteristics on placebo analgesia induced by observational learning

**DOI:** 10.1371/journal.pone.0243996

**Published:** 2020-12-16

**Authors:** Elżbieta A. Bajcar, Karolina Wiercioch-Kuzianik, Dominika Farley, Wacław M. Adamczyk, Ewa Buglewicz, Przemysław Bąbel

**Affiliations:** 1 Pain Research Group, Institute of Psychology, Jagiellonian University, Kraków, Poland; 2 Department of Kinesiotherapy and Special Methods in Physiotherapy, The Jerzy Kukuczka Academy of Physical Education, Katowice, Poland; University of Vienna, AUSTRIA

## Abstract

Previous studies have proved that observational learning can induce placebo analgesia, but the factors that influence observationally induced placebo analgesia have not yet been extensively examined. The primary goal of this study was to investigate the effect of information about the role that the observed person (model) plays in the experiment on the magnitude of the observationally induced placebo effect. This study also examined the contribution of the observer’s empathy, conformity and fear of pain to the placebo analgesia induced by observational learning. The effects induced in two experimental groups and one control group were compared. Participants in the experimental groups observed a model introduced as either another participant taking part in the study or a coworker of the experimenter. The model rated the intensity of pain induced by electrocutaneous stimuli preceded by color stimuli. One-half of all participants watched a model rating pain stimuli preceded by the color orange as higher than stimuli preceded by the color blue; for the other half, the ratings were the opposite. There was no observation in the control group. Subsequently, all participants received pain stimuli of the same intensity preceded by orange and blue stimuli and rated the intensity of the experienced pain. Placebo analgesia was found in both experimental groups. However, the way the observed model was introduced to participants did not affect the magnitude of placebo analgesia. Thus, the study showed that the role played by the model is not crucial for observationally induced placebo analgesia. The examined observer’s individual characteristics did not predict the magnitude of placebo effect.

## Introduction

Human beings are social beings and learn not only from direct experience but also vicariously by observing the experiences of other people [1]. Experimental studies have provided strong evidence that observational learning is one of the explanatory mechanisms of phenomena that are common in clinical settings, i.e. placebo analgesia [2–5] and nocebo hyperalgesia [3, 6–8]. However, despite the strong evidence that observational learning can induce placebo effects (for review see [9, 10]), the factors influencing the magnitude of analgesia or hyperalgesia induced in this way have not yet been fully elucidated.

According to the social learning theory, the characteristics of the model and the observer may contribute to the effectiveness of observational learning [1, 11]. The attributes of the model which could have an impact on placebo effects have been investigated in two studies. The study by Świder and Bąbel [6] showed that observation of a male model resulted in greater nocebo hyperalgesia than observation of a female model. It has also been demonstrated that observation of a videotaped model and a live demonstrator may be equally effective in inducing placebo analgesia [4]. These studies focused on examining the most vivid attributes of the model, such as sex or physical presence. The major goal of the current study was to investigate whether introducing the model as either another participant taking part in the study or a coworker of the experimenter would affect the magnitude of observationally induced placebo analgesia. Thus, in this study, the attributes of the model were not directly observable, but participants had to derive them from the provided information. From a theoretical perspective, modeling is a process of social comparison [12, 13]. The observers pay attention more willingly to the models that are similar to them or that are perceived by them as similar [14]. Moreover, the model that is similar to the observer influences the observer's self-efficacy, and thereby the learning outcomes [15]. Therefore, it has been hypothesized that a model presented as another participant would be more effective in shaping the observer's response to a placebo and induce greater effect than a model presented as a coworker of the experimenter.

Research on observer’s attributes that influence placebo effects is rare and rather inconclusive. A few previous studies suggest that the observer’s empathy may affect placebo effects induced by observation of a live model [2, 4, 6] but the role of empathy has not been confirmed in all studies [5]. Even less is known about the role of the observer’s conformity, defined as the change in one's behavior to match the responses to others [16]. It has been shown that pain reports provided by other people were able to modify the pain sensations of study participants, and that conformity was not involved in this effect [17]. However, in that study, neither observational learning nor placebo was applied, so its results do not answer the question of whether conformity contributes to observationally induced placebo effect. Some previous studies also showed that placebo effects may be linked to fear of pain [18, 19], however this was not always the case [7]. Thus, the second aim of the study was to clarify the previous results by investigating the effects of empathy, conformity and fear of pain on the magnitude of the placebo effect induced by observational learning.

## Materials and methods

### Design

In this study, three groups were tested: 1) demonstrator group, 2) co-participant group, and 3) control group. In the demonstrator and co-participant groups, during the observation phase the participants observed a model who had allegedly been subjected to electrocutaneous stimulation. Before each electrocutaneous stimulus, one of two colors was presented on the screen. The model simulated responses to electrocutaneous stimuli preceded by one of the colors (which served as placebo) as less painful than those preceded by the other. The model rated aloud the intensity of pain, while the participant was instructed to write down the ratings and colors which preceded them. In the demonstrator group, participants were informed that the model was a coworker of the experimenter and that she would present how to use the pain intensity scale. In the co-participant group, participants were informed that the model was another participant and that they were taking part in the study together. There was no observation phase in the control group. In the testing phase, all participants received the same number of electrocutaneous stimuli of the same, individually adjusted intensity, and rated the intensity of experienced pain.

### Participants and models

#### Participants

A total of 96 healthy volunteers, including 60 women (62%), aged 22 ± 2,67 participated in the study. Participants were recruited by announcements on classified advertisement websites and social media and received financial compensation for their participation. Participants were randomly assigned to one of the three groups: demonstrator group (N = 31), co-participant (N = 32), and control group (N = 33).

All participants were physically and mentally healthy, free of pain and were not taking any type of pain medication. None of them took part in any previous pain-related studies. The exclusion criteria were: 1) age below 18 or over 35; 2) pain during the last month; 3) taking any regular medication including non-prescription drugs; 4) using illegal drugs; 5) history of any respiratory, circulatory, neurological, musculoskeletal and/or psychiatric disorders; 6) current symptoms of depression and/or anxiety. The criteria were based on those proposed by Gierthmühlen and collaborators [20]. The Hospital Anxiety and Depression Scale (HADS) [21] was used to exclude people with anxiety or depression symptoms. People above 35 years old were excluded to increase homogeneity of the group as the evidence shows that pain perception is changing throughout the lifespan [22–24].

Participants were informed that they were participating in a study on pain mechanisms and that they would receive painful electrocutaneous stimulation. Having read the description of the experimental procedures, participants gave their informed, written consent to participate in the study. They were also informed that they could withdraw their consent at any time without providing a reason. When the study was completed, all participants were fully debriefed and informed about the actual aim of the experiment. The study protocol was approved by the Research Ethics Committee at the Institute of Psychology, Jagiellonian University, Kraków, Poland.

#### Models

In the demonstrator and co-participant groups, two trained female coworkers in their twenties served as models. The models were counterbalanced in such a way that half of all participants of either sex in each experimental group observed one model, and the other half observed the other model. The only difference was the way the models were introduced to the participants from experimental groups (‘coworker of the experimenter’ versus ‘another participant’). In both the co-participant and the demonstrator group, the model was already present in the laboratory when the participant entered. In the co-participant group, she was sitting in front of the computer with an electrode attached pretending to be undergoing the procedure. In the demonstrator group, the model was standing next to the experimenter until she was asked to ‘demonstrate the procedure’.

### Sample size

Sample size calculation was performed based on the data derived from the experiment where observational learning was used to induce placebo effects [6]. In order to detect a significant difference in pain intensity (mean value of 1.12 on 0–10 NRS scale, effect size d = 0.79) between the experimental and control group for the pain intensity outcome, it was estimated that a minimum sample of 21 subjects was required (alpha = 0.05, 80%, between-group comparison). However, to account for potential dropouts and to increase the power to detect the effect, a total of 96 participants (more than 30 per group) were examined. Power calculation was performed *a priori* by G*Power (G*Power 3.1.9.2 statistical software) [25].

### Stimuli

Electrocutaneous pain stimuli were delivered to the inner side of the non-dominant forearm of the participant through 2 durable stainless-steel disk electrodes that were 8 mm in diameter with 30 mm spacing. The electrocutaneous stimuli were square pulses with a duration of 200 μs, delivered by a Constant Current High Voltage Stimulator (Digitimer, Welwyn Garden City, England, Model DS7AH). The intensity of the electrocutaneous stimuli was set individually for each participant according to the calibration procedure (see: Procedure).

The pain stimuli were preceded by the presentation of color stimuli (orange or blue) displayed in full-screen mode on a computer screen (17 inches, resolution 1280x1024) placed in front of the participant (or the model) at a distance of approximately 50 cm. Color slides were presented according to a predetermined pseudorandom sequence.

The experimental procedure was fully automatized with PsychoPy software [26]. This software integrates stimuli application and data collection in real time.

### Measures

Pain intensity ratings were obtained by means of an 11-point numeric rating scale (NRS) ranging from 0 = “no pain” to 10 = “the most intense pain that is tolerable”. Each participant was also asked to complete four questionnaires measuring relevant psychological traits: 1) the Polish version of *Interpersonal Reactivity Index* (SWE) [27], adapted from the Interpersonal Reactivity Index (IRI) [28] which measures differences in dispositional empathy using three subscales, *Perspective Taking*, *Personal Distress* and *Empathic Concern*; 2) *The Gudjonsson Compliance Scale* (GCS) [29] which measures the tendency to conform to requests made by others in order to please them or to avoid conflict and confrontation; 3) *Measure of Susceptibility to Social Influence* (MSSI) [30], Polish adaptation [31], which measures the tendency to yield to the social influence, i.e. independence (*Principled Autonomy)*, conformity/compliance (*Social Adaptability)*, and anticonformity (*Social Friction)*, 4) *The Fear of Pain Questionnaire* (FPQ-III) [32], which measures the tendency to react with fear on pain and consists of three subscales, *Severe Pain*, *Minor Pain*, *Medical Pain*.

### Procedure

**Fig 1:**
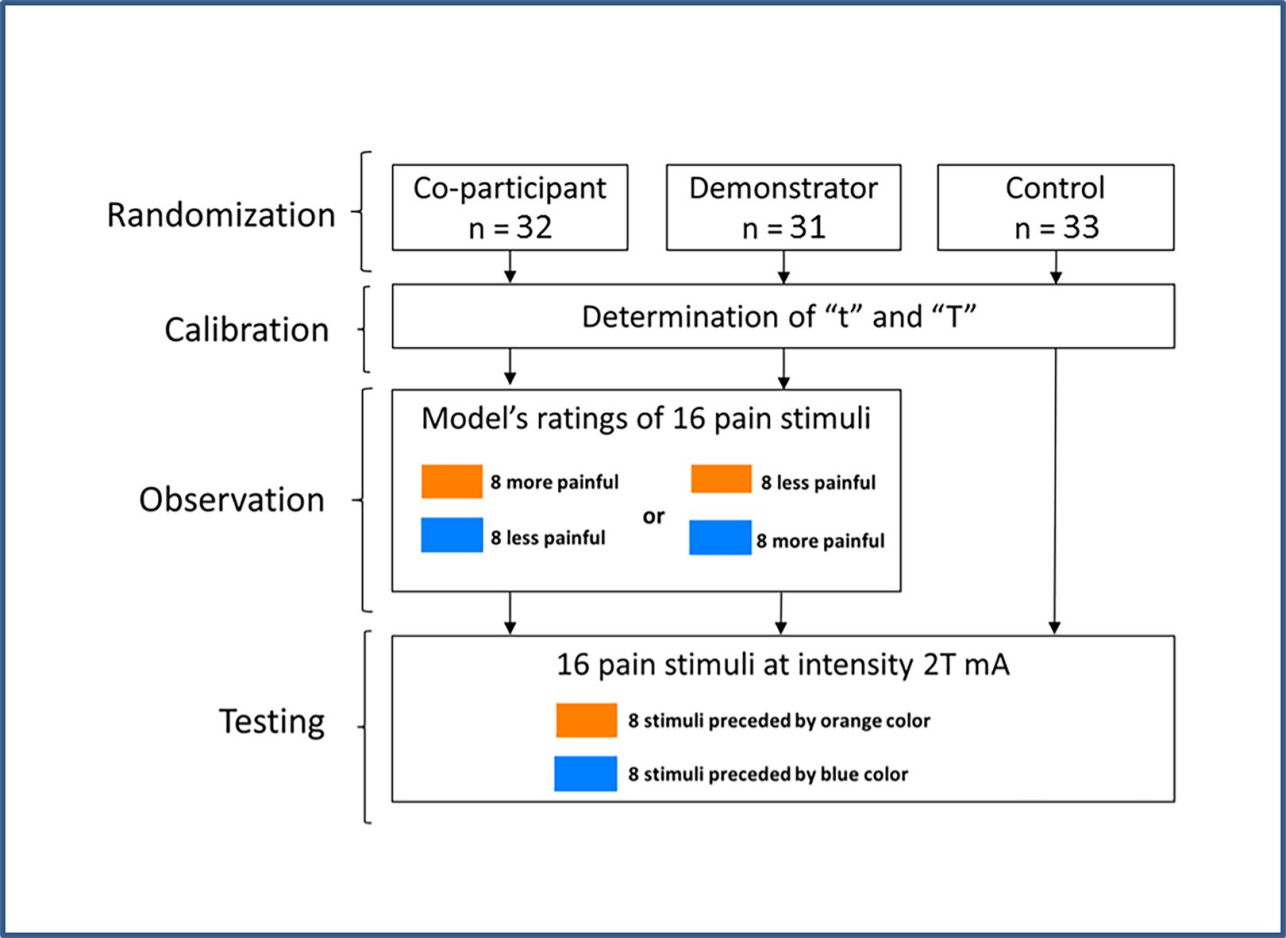
Study design. The study consisted of two experimental groups (co-participant group and demonstrator group) and one control group. In the co-participant and demonstrator groups, participants observed a model (presented to the participants as another person taking part in the study or as a coworker of the experimenter, respectively) who rated electrocutaneous stimuli preceded by one of the colors as less painful than those preceded by the other. In the control group, there was no observation phase. In the testing phase, all subjects received 16 pain stimuli of the same individually adjusted intensity.

The experiment consisted of three phases: calibration, observation, and testing. All groups underwent calibration and testing phases, while the observation phase was used in only two experimental groups, i.e. the demonstrator and co-participant groups (Fig 1).

#### Calibration phase

During the calibration phase, nonpainful tactile sensation thresholds (t) and pain thresholds (T) were determined for each participant. The calibration procedure consisted of two ascending series of electrocutaneous stimuli in increments of 0.5 mA (the interstimulus interval was 5 sec), starting from 0 mA. The intensities of the stimuli were gradually increased until participants detected the first nonpainful tactile sensation (t) and then until the sensations became painful (T), which was clearly stated by the participant. The mean of the two measurements of pain thresholds was calculated and the result subsequently doubled in order to establish the stimulus intensity (2T mA) that was used throughout the experiment

#### Observation phase

The observation phase took place only in the demonstrator and co-participant experimental groups. During the observation phase, participants observed the model rating 16 pain stimuli aloud using an NRS scale. Half of the stimuli were rated by the model as more painful (range 7–9 on the NRS), and the other half were rated as less painful (range 2–4 on the NRS). Half of the participants were randomly assigned to a condition in which the model rated stimuli preceded by orange and blue as more painful and less painful, respectively; the other half were in the opposite condition where stimuli preceded by orange and blue color were rated as less painful and more painful, respectively. This way, participants had an opportunity to associate each of the colors (blue or orange, depending on a random assignment) with either high or low pain ratings. In order to ensure that participants paid attention to both the pain ratings and the colors accompanying the pain stimuli, they were asked to write down the color of the slide and the model’s rating in a special form [5, 6]. In fact, no pain stimuli were administered to the model.

Participants in the demonstrator group were informed that they were observing a coworker of the experimenter who would show them how to use the pain rating scale, whereas participants in the co-participant group were informed that they were observing another participant of the study. There was no observation phase in the control group. In this group, the testing phase followed the calibration phase without delay.

#### Testing phase

Participants from all three groups took part in the testing phase. During this phase, each participant received 16 pain stimuli at intensity 2T mA, preceded by 8 orange or 8 blue slides presented in a pseudorandom order. A single trial consisted of 1) a color slide displayed for 9 seconds, 2) a pain stimulus applied 7 seconds after the beginning of the trial with the color slide still visible, and 3) the NRS scale displayed on the same color slide after the pain stimulus was applied.

#### Manipulation check

After the testing phase was, participants were asked to describe what in their opinion the real goal of the study was. Subsequently, the participants were asked a series of questions (answers yes/no): 1) whether the presented colors were linked to pain intensity, 2) whether the presented colors were linked to the pain ratings provided by the model, 3) whether observing the model facilitated subsequent pain ratings. Afterwards, they completed the psychological traits questionnaires.

### Data reduction and statistical analysis

Participants rated pain stimuli preceded by two colors. The color preceding the pain stimuli rated by the model as less painful is further referred to as color_LOW_, while the color preceding the stimuli rated by the model as more painful is referred to as color_HIGH_. This distinction into color_HIGH_ and color_LOW_ reflects the two within-subject conditions to which participants were exposed during the experiment. The former refers to the color preceding the models’ pain ratings of higher intensity, while the latter refers to the color preceding pain ratings of lower intensity. In the control group, the blue-control and orange-control conditions reflect the participants’ pain ratings following painful stimuli presented with respective colors. For the purpose of the analysis, the term “placebo analgesia” was introduced to reflect the magnitude of the difference between the two conditions, i.e. color_HIGH_ and color_LOW_.

Descriptive statistics (means and SDs) were calculated for the following variables: the tendency to conform measured by GCS, susceptibility to social influence measured by MSSI, empathy measured by IRI, fear of pain measured by FPQ-III, age, height, body mass, tactile and pain thresholds. To investigate if there were any between-subject differences in the level of these variables, a one-way ANOVA design (Bonferroni test) with “group” (co-participant, demonstrator, and control group) as an independent variable was conducted.

The main analysis was performed on participants' NRS pain ratings using a repeated-measures analysis of variance (ANOVA) design, with “group” (co-participant, demonstrator, and control group) and “stimulus” (color_HIGH_ and color_LOW_) as a within-subjects factor. The *F*-tests were followed by planned comparison tests between NRS pain ratings associated with color_HIGH_ and color_LOW_ in each of the groups. Then, differences between color_HIGH_ versus color_LOW_ in the co-participant and demonstrator groups were compared with the difference between NRS pain ratings associated with the blue-control and orange-control conditions in the control group. Similarly, the difference between color_HIGH_ and color_LOW_ in the demonstrator group was compared to that difference in the co-participant group.

Pearson product-moment correlation coefficients (*r*) were calculated to explore the relationship between participants’ and model’s pain ratings and to investigate the relationship between placebo analgesia and questionnaires’ scores (GCS, MSSI, FPQ-III and IRI). To explore if there were any differences in in the magnitude of placebo analgesia between participants from the experimental groups who answered “yes” or “no” to each of the manipulation check questions, a two-way ANOVA design was conducted with “group” and “answer” (yes, no) as between-subject factors and the difference between color_HIGH_ versus color_LOW_ as a dependent variable.

The alpha level was set at 0.05 for the rejection of the null hypothesis in all the statistical analyses. Bonferroni correction was used to adjust control for multiple comparisons (adjusted alpha 0.016(6)). All the analyses were conducted using STATISTICA data analysis software, version 12 (StatSoft Inc., Tulsa, OK, USA).

## Results

The analyzed sample included 96 participants divided into three groups. The one-way ANOVA revealed that there were no differences between the groups (co-participant, demonstrator and control) in age, height, body mass, tactile threshold, pain threshold, IRI empathic concern, IRI personal distress, IRI perspective taking, MSSI principled autonomy, MSSI social adaptability, MSSI social friction, tendency to conform measured by the GCS scale, and fear of pain measured by FPQ-III. The characteristics of the participants in each group are presented in Tables 1 and 2.

**Table 1:** Descriptive statistics for age, weight, sensation and pain thresholds, and pain ratings—means and standard deviations.

Group	N	Age	Height (cm)	Body mass (kg)	t (mA)	T (mA)	Color_HIGH_	Color_LOW_
1	31	21.59 ± 2.16	172.24 ± 11.05	67.28 ± 13.76	3.06 ± 1.27	15.69 ± 11.31	5.88 ± 1.19	5.09 ± 1.49
2	32	22.25 ± 3.48	171.07 ± 8.97	67.14 ± 17.79	2.97 ± 1.3	14.55 ± 11.06	5.29 ± 1.43	4.7 ± 1.56
3	33	22.43 ± 2.2	174.14 ± 12.64	70.69 ± 13.15	3.33 ± 1.16	16.61 ± 12.44	3.97 ± 2.22	3.97 ± 2.2

**Abbreviations:** 1—demonstrator group; 2—co-participant group; 3—control group; t—sensation thresholds; T—pain threshold; Color_HIGH_ and Color_LOW_—pain ratings.

**Table 2:** Descriptive statistics for personality variables—means and standard deviations.

Group	N	GCS	IRI (EC)	IRI (PD)	IRI (PET)	MSSI (PA)	MSSI (SA)	MSSI (SF)	FPQ
1	31	7.55 ± 3.0	38.42 ± 7.27	23.58 ± 4.54	33.94 ± 5.54	55.45 ± 8.59	33.03 ± 7.35	26.16 ± 4.40	72.59 ± 16.19
2	32	8.53 ± 3.34	36.69 ± 9.63	22.50 ± 5.74	32.78 ± 5.53	53.69 ± 7.51	34.25 ± 7.25	25.13 ± 5.02	73.04 ± 12.24
3	33	8.06 ± 3.34	38.09 ± 7.63	21.06 ± 5.02	35.85 ± 4.04	56.79 ± 7.11	32.18 ± 6.89	25.27 ± 4.30	72.96 ± 17.83

**Abbreviations:** 1—demonstrator group; 2 –co-participant group; 3—control group; GCS—The Gudjonsson Compliance Scale; IRI—Interpersonal Reactivity Index: IRI (EC)—Empathic Concern; IRI (PD)—Personal Distress; IRI (PET)—Perspective Taking; MSSI—Measure of Susceptibility to Social Influence: MSSI (PA)—Principled Autonomy; MSSI (SA)—Social Adaptability; MSSI (SF)—Social Friction; FPQ—Fear of Pain Questionnaire.

### Main analysis

The repeated measures ANOVA on the NRS pain ratings revealed a statistically significant main effect for “stimulus” (*F*_(1,93)_ = 22.68, *p* < 0.001, *η*^2^_*p*_ = 0.20) and “group” (F_(2,93)_ = 6.97, p < 0.01, *η*^2^_p_ = 0.13) and a statistically significant “stimulus” × “group” interaction (*F*_(2,93)_ = 6.08, *p <* 0.01, *η*^2^_*p*_ = 0.12)

**Fig 2:**
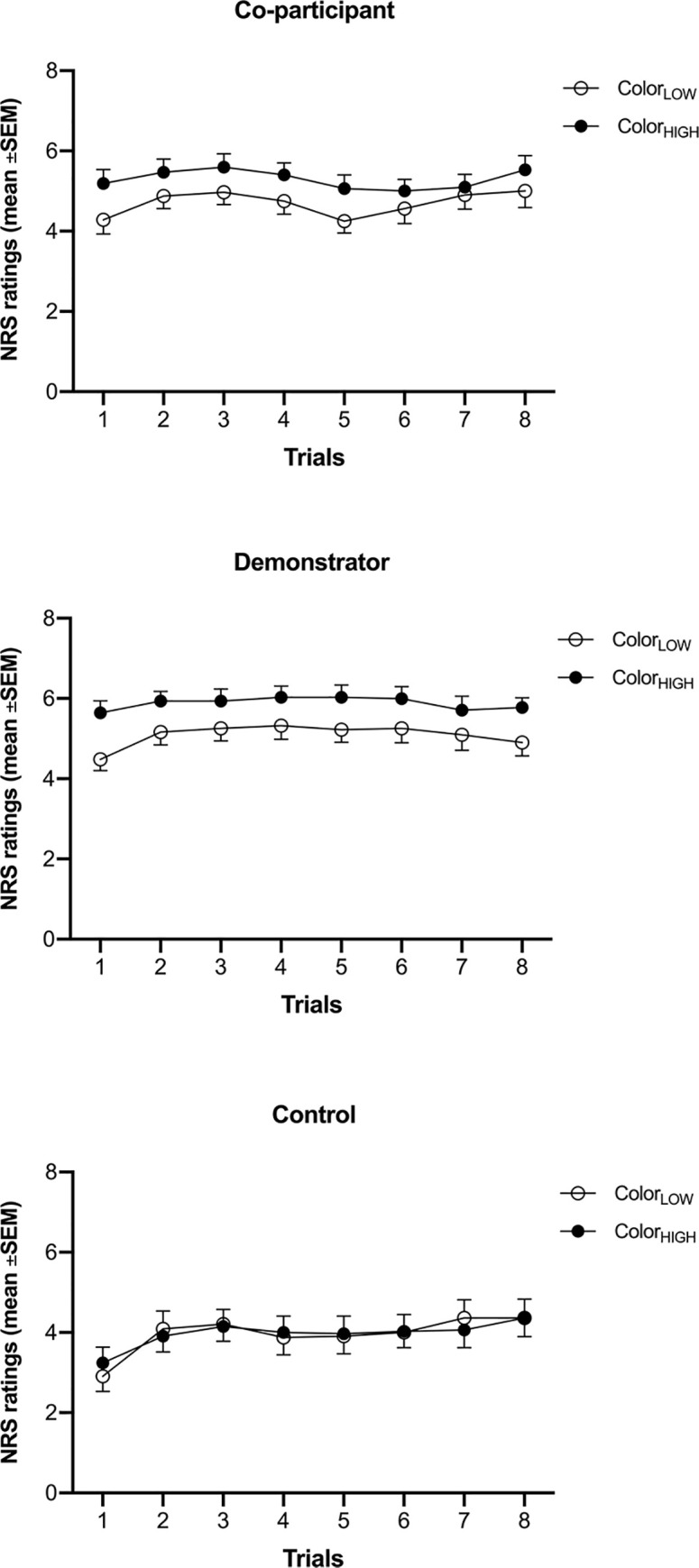
Pain ratings during the testing phase in co-participant group (A), demonstrator group (B) and control group (C). The figure depicts mean pain intensity ratings for stimuli preceded by color_LOW_ (i.e. the color preceding pain stimuli rated by the model as less painful) and color_HIGH_ (i.e. the color preceding stimuli rated by the model as more painful) in two experimental groups and preceded by color_1_ (i.e. blue or orange) and color_2_ (i.e. orange or blue) in the control group. **Abbreviations:** NRS–Numeric Rating Scale; SEM–Standard Error of the Mean.

Within-group planned comparison tests on pain ratings associated with color_HIGH_ versus color_LOW_ showed that observing a model had a significant effect on participants’ pain ratings in both the demonstrator (*F*_(1,93)_ = 21.60, *p* < 0.001, *η*^2^_*p*_ = 0.19) and co-participant group (*F*_(1,93)_ = 12.45, *p* < 0.01, *η*^2^_*p*_ = 0.12). Participants in both experimental groups experienced less pain when electrocutaneous stimuli were preceded by color_LOW_ compared to the condition in which electrocutaneous stimuli were preceded by color_HIGH_. Thus, placebo analgesia was found. There was no significant difference in pain ratings between the orange-control and blue-control conditions in the control group (*F*_(1,93)_ = 0.00, *p* = 1.0, *η*^2^_*p*_ = 0.00), indicating that the color of the stimuli with no previous observation of a model had no effect on pain ratings (Fig 2).

**Fig 3:**
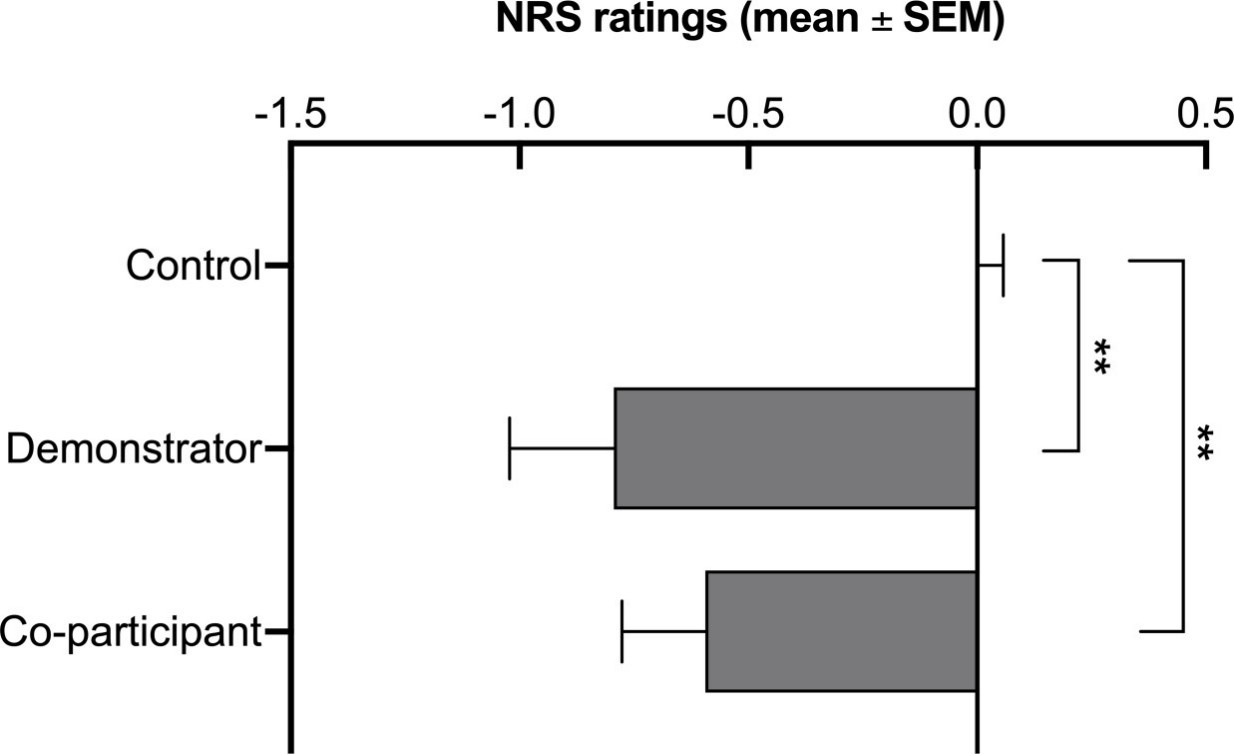
The magnitude of placebo analgesia. The figure depicts differences in pain intensity ratings between the co-participant group, demonstrator group and control group. **Abbreviations:** NRS–Numeric Rating Scale; SEM–Standard Error of the Mean.

The between-group planned comparison of the magnitude of the difference in pain ratings between the color_HIGH_ versus color_LOW_ conditions in the demonstrator group compared with the difference in pain ratings between orange-color and blue-color stimuli from the control group showed a significant effect (*F*_(1,93)_ = 11.14, *p* < 0.01, *η*^2^_*p*_ = 0.11). A similar comparison between the control and co-participant group also revealed a significant difference in pain ratings (*F*_(1,93)_ = 6.32, *p* < 0.05, *η*^2^_*p*_ = 0.04), indicating that in both groups, i.e. demonstrator and co-participant, participants experienced less pain in the color_LOW_ condition compared to the control group. However, the demonstrator and co-participant groups did not differ significantly in the magnitude of the difference in pain ratings between the color_HIGH_ versus color_LOW_ conditions (*F*_(1,93)_ = 0.70, *p* = 0.41, *η*^2^_*p*_ = 0.01), indicating that observationally induced placebo analgesia was similar in both groups (Fig 3).

Differences in the magnitude of placebo analgesia were detected when participants from experimental groups were split in terms of how they answered two of the three manipulation check questions. Namely, declaring that there was a relationship between the presented colors and pain stimuli had an influence on the placebo analgesia as reflected in a significant main effect of "answer" (*F*_(1,59)_ = 5.83, *p* = 0.02, *η*^2^_p_ = 0.09) but not significant "group" x "answer" interaction (*F*_(1,59)_ = 0.17, *p* = 0.68, *η*^2^_p_ = 0.003), indicating that those who answered positively experience stronger placebo effect. Similar trend in the data was found when analyzing answers to the question about the link between colors and model’s pain ratings: significant main effect of "answer" (*F*_(1,59)_ = 6.03, *p* = 0.02, *η*^2^_p_ = 0.09) and not significant "group" x "answer" interaction (*F*_(1,59)_ = 0.01, *p* = 0.93, *η*^2^_p_ < 0.001) indicate that stronger effect was observed in those participants who answered positively. Nevertheless, no differences in placebo analgesia were detected when participants from experimental groups were split in terms of how they answered the question about the facilitatory role of the observational phase ("answer": *F*_(1,59)_ = 2.83, *p* = 0.10, *η*^2^_p_ = 0.05, "group" x "answer" interaction: *F*_(1,59)_ = 1.83, p = 0.18, *η*^2^_p_ = 0.03).

### Correlations

Correlational analyses revealed that there was no significant relationship between the model’s and participants’ pain ratings in both the demonstrator (color_LOW_: *r* = -0.11, *p* = 0.558, color_HIGH_: *r* = -0.10, *p* = 0.607) and co-participant group (color_LOW_: *r* = 0.09, *p* = 0.645). (color_HIGH_: *r* = -0.19, *p* = 0.322). The lack of correlations between participants' and models' pain ratings indicates that participants' ratings represented their actual pain experience over those remembered as a result of observation of models' ratings.

The magnitude of placebo analgesia in the demonstrator group was not significantly correlated with IRI empathic concern (r = 0.05, p = 0.79), IRI personal distress (r = 0.20, p = 0.31), IRI perspective taking (r = 0.31, p = 0.11), MSSI principled autonomy (r = -0.14, p = 0.46), MSSI social adaptability (r = 0.324, p = 0.21), MSSI social friction (r = -0.35, p = 0.07), tendency to conform measured by GCS scale (r = 0.22, p = 0.24), and fear of pain measured by FPQ (r = -0.26, p = 0.17). Similar results were found in the co-participant group in relation to IRI empathic concern (r = -0.14, p = 0.47), IRI personal distress (r = -0.04, p = 0.84), IRI perspective taking (r = 0.08, p = 0.69), MSSI principled autonomy (r = 0.16, p = 0.42), MSSI social adaptability (r = -0.06, p = 0.75), MSSI social friction (r = -0.19, p = 0.34), tendency to conform measured by GCS scale (r = 0.13, p = 0.50), and fear of pain measured by FPQ (r = 0.14, p = 0.47).

## Discussion

A significant difference between placebo- and non-placebo pain ratings was found in participants who had previously observed a model rating pain stimuli preceded by a placebo stimulus as significantly less painful than pain stimuli preceded by a non-placebo stimulus. This result is in line with previous studies (for review see [9, 10]), and provides further evidence that placebo analgesia can be induced by observational learning.

The aim of the current study was to investigate the conditions under which observational learning can be most effective in shaping placebo effects. Previous studies in social psychology have shown that vivid and observable characteristics of the model, e.g. sex [33–35] or race [33, 36, 37], affect the effectiveness of social learning. It is also worth noting that the presence of contextual cues or information that direct the observer’s attention to attributes of the model, e.g. social status [38, 39] or competencies [38, 40–42], have also an effect on the learning process. This leads to the conclusion that not only directly observable physical characteristics but also information about the model derived from the context in which the model is observed can influence learning effects.

To the best of our knowledge, only two studies have been conducted to examine the role of directly observable characteristics of the model in shaping observationally induced placebo effects [4, 6]. The effect of the information about the model's attributes that are not directly observable on the magnitude of placebo effects has not yet been investigated. Although the results of two previous studies showed that observation of a model introduced as another participant can elicit placebo effects [5, 6], it is not clear how the model was introduced in other studies [2–4, 7, 8]. Based on these studies, it cannot be determined how the information that the model is a coworker of the experimenter influences placebo analgesia. The current study is the first to compare placebo effects induced by observing a model introduced as another participant of the study and as a coworker of the experimenter.

Previous studies have shown that people, even when assigned to a group arbitrarily, tend to see themselves as more alike [43, 44] and perceive the group members as a valid source of information [44]. Therefore, it was hypothesized that pain reports provided by a model perceived as another participant would cause a greater placebo effect than those provided by the experimenter’s coworker, but the obtained results did not confirm this hypothesis. It seems that in situations associated with the high probability of aversive events, people attempt to utilize all available cues that allow them to predict noxious stimulation. In the controlled experimental situation, the behavior of the model was the only source of information about the upcoming pain. Thus, it appears that the model, regardless of whether introduced as another participant or as a coworker of the experimenter, was perceived as a valid source of information about an aversive event. This conclusion can be also supported by results of the study on observationally induced placebo analgesia, in which effects elicited by different types of placebos were compared [5]. It was shown that placebo analgesia of a similar magnitude was induced regardless of whether different colors or geometric shapes were used as placebos. Thus, the current study provides further support that observational learning is effective in inducing placebo analgesia–it works regardless of the way in which the model is presented.

Interestingly, although participants in both experimental groups rated the pain stimulus as more or less intense depending on preceding color, their pain ratings were generally higher than those provided by the participants who did not observe the model in the course of the experiment. It seems that observing a model experiencing pain might influence participants’ sensitivity to pain, which is in line with findings from other pain studies [45–48]. Moreover, the model in our study rated the intensity of pain relatively high, which could have significantly affected the participants' pain perception.

The studies on the observer’s characteristics in relation to placebo effects gave inconclusive results. One of the most examined individual characteristics of the observer is empathy [2, 4–8]. The results obtained in the current study showed that empathy did not contribute to the magnitude of observationally induced placebo effects. A similar result was obtained in one previous study where a model was observed directly [5] and in studies where video recording was used instead of a live model [4, 7, 8]. However, in most of the previous studies the correlation between empathy and the effects of modeling was at best moderate [2, 4, 6]. Moreover, in most of them only one of the dimensions of empathy, i.e. empathic concern, was involved in shaping placebo effects [2, 4]. These data, as well as data from the current study, suggest that dispositional empathy can be an important characteristic associated with observational learning, but not a pivotal one.

Studies in social psychology have shown that instructions or information provided by other people can lead to conformity [16, 49–51] which manifests itself by matching responses of a given individual to these presented or suggested by others. The result of the current study did not show that observer’s conformity was involved in observationally induced placebo analgesia. This result is in line with the findings of Koban and Wager [17]; however, unlike our research, in their study participants did not observe a model experiencing pain but they received information on how other people rated the intensity of pain. Regardless of methodological differences, conformity was not involved in both placebo analgesia obtained in the current study and analgesic effect obtained by Koban and Wager’s [17]. Moreover, no significant correlations were found between pain ratings provided by the participants and by the model in the current study. This indicates that pain information provided by the model had an effect on participants’ pain experiences rather than on their ratings.

Furthermore, no correlation between the observer’s fear of pain and placebo effect has been found. This result is in line with findings obtained in the previous study where the videotaped model was presented to elicit nocebo hyperalgesia [7]. It seems that further studies are needed to establish the role of fear of pain in the formation of placebo analgesia induced by direct observation of a model.

There are a few advantages of the current study that should be acknowledged. First, this is the first study to investigate experimentally whether verbally provided information about the role that the model plays in the experiment may influence the magnitude of observationally induced placebo analgesia. Moreover, both men and women were involved in this study, while in most of the previous studies on placebo effects induced by observational learning, only women participated [2, 4, 5, 7, 8]. Second, color was the only placebo in the current study; no interventions were implemented (i.e. fake electrodes or creams), the use of which would suggest the analgesic effect. Thus, the placebo analgesia found in this study was induced by pure observational learning, without any suggestions. It should be noted here that the effect obtained in this study can be classified as the placebo effect despite the fact that it was induced without the use of placebo intervention in the form of a sugar pill or fake ointment. According to Miller and Kaptchuk [52], the placebo effect is not the result of a specific intervention, but it is produced by the context surrounding the treatment. Thus, each stimulus that accompanies the changes in pain responses of the model has the potential to become a placebo stimulus and induce in the observer placebo analgesia. This view is shared by the large body of theorists and researchers [53]. Third, the color stimuli were counterbalanced, and the control group was included in the study design. Thus, the results are not biased by the colors used, which is important in the light of previous findings that showed that colors have an effect on pain perception [54].

Some limitations of the current study need to be acknowledged and addressed in future studies. In this study, acute pain was induced by the application of electrocutaneous stimuli, thus the results of the study should be generalized to clinical pain with caution. Moreover, the variables rely on self-reports, but this is also the case in most of the previous studies on observationally induced placebo effects [4–8]. Similarly to other studies on observationally induced placebo effects [2, 4–8], in the current study expectancies were not measured. If measured, they could have helped in specifying the mechanism of observationally induced placebo analgesia.

The results of this study not only extend the knowledge on the conditions that contribute to the magnitude of the observationally induced placebo effect, but they also lead to an important methodological implication. In most of the previous studies, participants did not receive any particular information about the model [2–4, 7, 8]. Only in two studies were participants explicitly informed that they were observing another participant being subjected to the same experimental procedure [5, 6]. Although placebo effects were induced in all the cited studies, it was not clear to what extent instructions provided to participants influenced their magnitude. The results obtained in this study showed that the information about the role played by the model is not crucial in the learning process and does not contribute significantly to the magnitude of placebo analgesia.

The results of this study also have important implications for clinical practice. This research suggests that the broadly defined social environment may affect individual pain experiences and shape pain behaviors. Not only other patients experiencing pain but also trained models may be a credible source of information concerning pain. Promoting pain-free behaviors by observing others who experience pain relief can be used as a complementary technique to standard pain-management programs. Considering the fact that observing others can induce also nocebo response, one of the main priorities is to provide individual care to patients.
